# Analysis of influencing factors and equity in health education for mobile populations based on random forest model: evidence from the China migrants dynamic survey

**DOI:** 10.3389/fpubh.2025.1570633

**Published:** 2025-05-08

**Authors:** Meng Han Jiang, Shan Yan Lei, Fang Yang

**Affiliations:** ^1^School of Humanities and Management, Zhejiang Chinese Medical University, Hangzhou, China; ^2^Department of Rehabilitation Medicine, The First Affiliated Hospital, Zhejiang University School of Medicine, Hangzhou, China

**Keywords:** mobile population, health education, influencing factors, equity, random forest model

## Abstract

**Background:**

Strengthening health education is an important measure to improve the health of mobile populations and a key objective of China’s basic public health services. Existing studies demonstrate that health education affects the health of mobile populations, but insufficient attention is paid to the importance of factors that influence health education. Moreover, few studies examine how these factors contribute to health education equity among mobile populations in China. Therefore, this study aims to reveal the importance of factors affecting health education based on a comprehensive understanding of mobile populations’ overall health education status. Furthermore, the contribution of these important factors to health education equity is analyzed to inform differentiated intervention strategies, thereby providing a reference for enhancing mobile populations’ health level and achieving equal access to basic public health services.

**Methods:**

This study utilized data from the 2018 China Migrants Dynamic Survey (CMDS), with a final sample of 103,910 participants after data cleaning. Chi-square tests were first conducted to examine differences in health education across various characteristics of the mobile population. The relative importance of influencing factors was then assessed using a random forest model, followed by key factor identification through LASSO regression. Subsequently, binary logistic regression was performed to quantify the effects of these key factors. Finally, concentration indices were calculated to identify these factors’ contributions to health education equity.

**Results:**

The self-assessed health status of China’s mobile population was good, with 81.89% reporting receipt of health education, while 18.11% had not received any health education. Seven key factors were identified as most influential in determining health education access among the mobile population: income, education, age, health record, scope of mobility, reason for mobility and gender. The health education concentration index of the mobile population was 0.0121, indicating a significant inequality in the utilization of health education services. Each important factor had a significant negative amplifying effect on health education equity among the mobile population.

**Conclusion:**

Health education among the mobile population requires further enhancement. Special attention should be directed toward vulnerable groups, including low-income individuals, the older adult, those with lower educational levels, and those with wider migration ranges. Moreover, the impact of key factors on health education equity among the mobile population should be carefully considered to improve health education equity.

## Introduction

Mobile population is a concept that emerged from the household registration system in China in the past 40 years (1). It refers to people aged 15 and above who have lived in the inflow area for more than 1 month and are not household members of their own district (city or county). According to the seventh national census data released by the Chinese government in 2021, the number of the mobile population reached nearly 380 million in 2020, representing an increase of 150 million from 2010 (2). The mobile population has the characteristic of “mobility,” which may cause social integration, policy neglect and other problems, and bring certain difficulties to social governance. Compared with other groups of people, the mobile population is a group that cannot be ignored, and the research results of scholars in different countries on the mobile population show that most of the mobile population have a low education level, mostly engaged in labor-intensive work, poor working environment and living conditions, which makes them face higher health risks (3, 4). At the same time, their physical and mental health problems are often neglected, resulting in inadequate social support and protection. Their access to public health services is lower than that of local residents (5, 6). Therefore, the health protection of the mobile population should receive greater attention.

Health education is an educational activity that promotes the acquisition of health knowledge, fosters health awareness, and encourages voluntary adoption of healthy behaviors and lifestyles among individuals or groups through information dissemination and behavioral interventions (7). Health education is not only one of the main contents of China’s basic public health service program (8), but also a strategic health care measure recognized worldwide (9). Effective health education is conducive to improving the health knowledge of individuals or groups, helping them to establish good health beliefs, and improving poor health-related behaviors (10). To enhance the health status of the mobile population, China has consistently prioritized their health education, positioning it as a crucial initiative to achieve equal access to basic public health services and promote health equity. The State Council of the Central Committee of the Communist Party of China (CPC) issued the “Healthy China 2023” Planning Outline in 2016, which emphasizes strengthening health education and actively promoting equal access to basic public health services for mobile populations. This aims to further reduce the disparities in health levels among populations and promote social equity (11).

With regard to health education for the mobile population, relevant studies can be summarized in the following three areas. First, in terms of the current situation, a study shows that the acceptance rate of health education for China’s mobile population is 73.1%. The primary content focuses on smoking control, maternal and child health care and eugenics, reproductive health and contraception, while mental health, tuberculosis prevention and treatment and prevention of occupational diseases are relatively lacking in the type of health education. The main delivery methods include publicity materials (paper, television programs, film and television), bulletin boards, public health counseling (12, 13). Other studies reveal that the level of health education received by urban and rural mobile populations in China is uneven, with urban mobile populations receiving higher levels of health education (14). Second, in terms of influencing factors, studies have pointed out that gender, age, education level, marital status, monthly household income, annual per capita GDP, occupation type, inflow time, inflow area, whether they suffer from chronic diseases, and whether they have had a health record can influence the level of health education of the mobile population (15–17). Third, in terms of fairness, current studies have paid more attention to the health status of the mobile population and the fairness of the utilization of public health services such as family doctor contracting and health records, but have paid less attention to the equity of health education (18, 19).

Current literature demonstrates extensive scholarly attention to health education among China’s mobile population, but there is still some room for enhancement. Firstly, current research generally employs descriptive statistics and logistic regression methods to analyze influencing factors, with little comparison of the relative importance of these factors. Secondly, the current research primarily focuses on the fairness of family doctor contracts and health records among China’s floating population, with limited studies addressing the fairness of health education service utilization.

To address the above deficiencies, this study takes health education for the mobile population as the research object. First, the current situation of health education for the mobile population is analyzed. Second, the Random Forest model is employed to rank the relative importance of factors and identify key variables. Third, regression analysis is conducted to explore the influencing factors of health education for the mobile population. Finally, the Concentration Index is applied to identify the key factors influencing the equity of health education. The findings are expected to inform the development of targeted health education interventions and promote equal access to basic public health services for mobile populations.

Compared with existing studies, this study offers several contributions: First, the study uses large-scale micro-survey data specifically targeting the migrant population in China, and the current situation of health education for the mobile population is comprehensively analyzed from multiple dimensions and levels. This helps to understand the health of the mobile population from the overall level. Second, current research on health education for the migrant population typically explores the independent effects of different factors or the interactive influences among them, but lacks a comparison of the relative importance of these variables. This study uses a random forest model to measure the importance of various influencing factors and identifies the most critical ones, which helps to provide a more accurate evaluation of these factors. Thirdly, this study focuses on the equity of health education in the utilization of public health services by the migrant population. This not only enriches existing analyses of equity among migrant populations but also provides data support and theoretical foundations for developing more targeted health education policies.

## Research methods

### Theoretical framework

**Figure 1:**
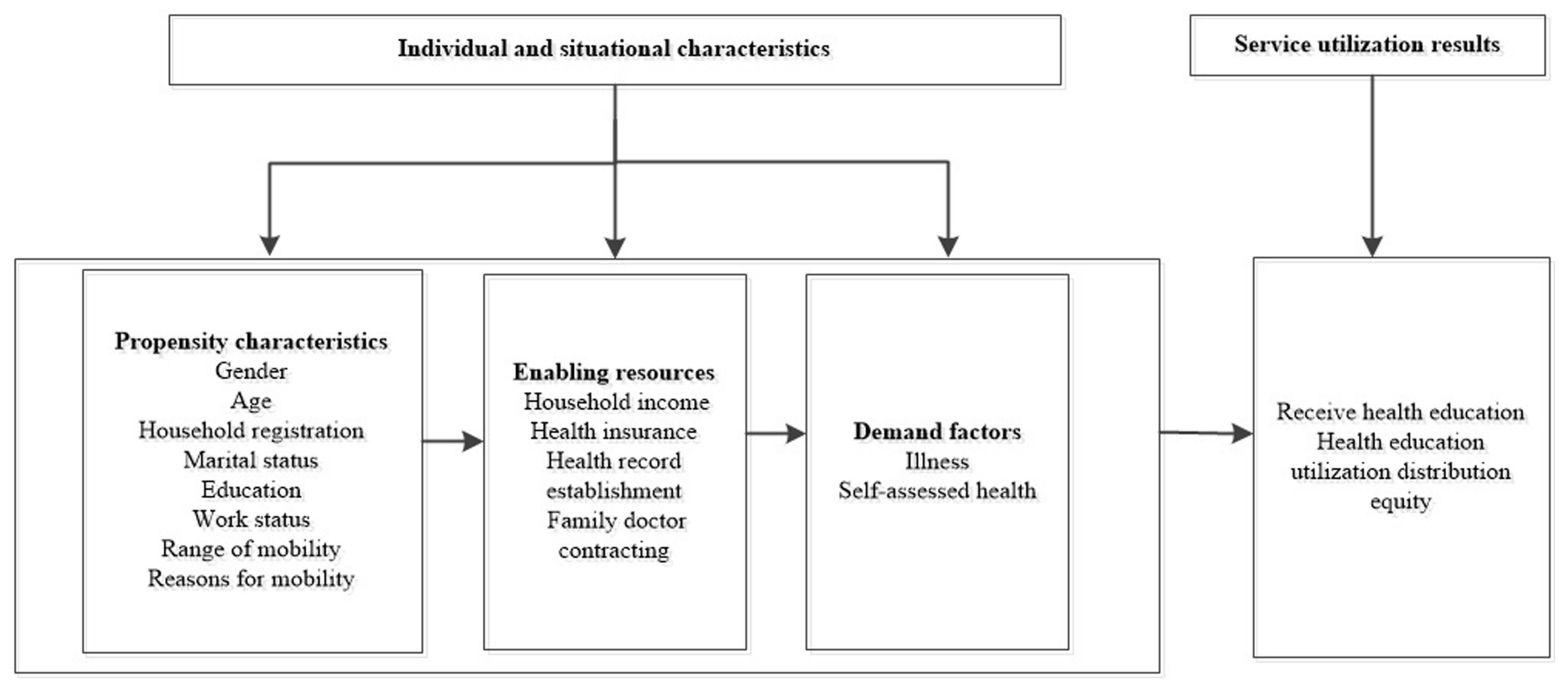
The health education service utilization behavior analysis framework for the mobile population based on the Anderson model.

The theoretical framework can further clarify the determinants of health education and the inequalities among the migrant population. Current theoretical frameworks related to healthcare utilization in academic literature include the Social Determinants of Health Action Framework (20), the Health Equity Model (21), and Anderson’s Behavioral Model of Health Service Utilization (22), among others. Among these, Anderson’s Behavioral Model of Health Service Utilization is a significant theoretical model for studying healthcare utilization behavior and has been widely applied in the healthcare research field (23, 24). Additionally, this model has been used in relevant studies to analyze the utilization of public health services by the migrant population (25). Therefore, this study adopts Anderson’s Behavioral Model of Health Service Utilization as its theoretical framework. Based on the characteristics of the migrant population and relevant literature (26, 27), the study constructs an analytical framework for the factors influencing health education service utilization among China’s migrant population, with a specific classification framework shown in Figure 1.

### Data source

The data for this study were obtained from the 2018 China Migrants Dynamic Survey (CMDS), which is an annual large-scale national mobile population sample survey conducted by the National Health Commission of China since 2009.The CMDS is considered to be a good representative sample, and has a small sampling error (28). The survey adopted a hierarchical, multi-stage, and proportionate-to-size PPS method for sampling, i.e., in the first stage, the townships (towns and streets) were selected according to the PPS method. In the second stage, village (neighborhood) committees were sampled within the selected townships (towns and streets) according to the PPS method. In the third stage, individual respondents were drawn within the selected village (neighborhood) committees. The survey also adopted strict methods to ensure data quality, including scientific design of questionnaires, training of surveyors, setting up survey supervisors to verify questionnaires, and quality checking by means of telephone callbacks.

The survey selects as its sample mobile population in 32 provincial administrative regions of China who are at least 15 years of age, have lived in their current location for more than 1 month, and are not household members of their own district (county or city). The survey covers basic demographic information on the mobile population and household members, the scope and tendency of mobility, employment and social security, income and expenditure, residence, basic public health services, marriage and family planning services, children’s mobility and education, and psycho-culture, which makes the survey a rich set of variables. A total of 152,000 samples of the mobile population were collected in this survey, and the final sample comprised 103,910 after processing the missing values and outliers of the data.

### Variables selection

#### Dependent variable

The core explanatory variable in this paper is health education. A 0–1 dummy variable was constructed based on the questionnaire: “Have you received any of the following types of health education in your current community/workplace during the past year?.” The answers to this question include occupational disease prevention and control, infectious disease prevention and control, reproductive health and maternal and child health, chronic disease prevention and control, mental health, self-help in public emergencies, and others. Referring to related studies (13, 29), this study assigns a value of 1 to the mobile population who received one or more types of education, and 0 to those who did not.

#### Independent variables

Based on the analytical framework for the utilization of health education services for mobile populations established in the theoretical foundation, the independent variables in this study were classified into three dimensions: (1) propensity characteristics: including gender, age, education, marital status, work status, scope of mobility, reasons for mobility and household registration; (2) enabling resources: including household income (in this study, data about household income were transformed into rankings within each province: < 20th percentile, 20th-39th percentile, 40th-59th percentile, 60th-79th percentile and ≥ 80th percentile for data analysis), health insurance, health record establishment and family doctor contracting; (3) demand factors: including self-assessed health and illness, with variable assignments detailed in Table 1.

**Table 1:** Variable selection and definitions.

Variables		Definition
Implicit variable	Health education	No = 0, Yes = 1
Independent variable	Gender	Female = 0, Male = 1
	Age	15–30 = 1, 31–45 = 2, 46–60 = 3, ≥61 = 4
	Educational attainment	Illiterate = 1, Elementary school = 2, Middle school = 3, High school = 4, University and above = 5
	Marital status	Not in marriage = 0, In marriage = 1
	Working conditions	No occupation = 0, Employed = 1
	Household registration	Rural = 1, Urban = 2
	Range of mobility	Cross-county within city = 1,Cross-city within province = 2, Cross-province = 3
	Reasons for mobility	Family = 1, Work = 2, Other = 3
	Household income ranking	Lowest (<20%) = 1, Lower (20–39%) = 2, Medium (40–59%) = 3, Higher (60–79%) = 4, Highest (≥80%) = 5
	Insurance location	Place of domicile = 1, Place of inflow = 2
	Whether one is sick or not	No = 0, Yes = 1
	Health level	Unhealthy = 1, Basically healthy = 2, Healthy = 3
	Availability of health records	No = 0, Yes = 1
	Whether the family doctor signed up	No = 0, Yes = 1

### Data processing

#### Random forest model

The random forest model not only allows for the comparison of the importance of various variables, which identifies relatively important ones, but also offers high accuracy, efficiency in testing, and stability, making the results more reliable (30). Compared to more complex models such as XGBoost, the advantages of the random forest model are as follows: (1) It provides a more intuitive analysis of feature importance and requires less adjustment. (2) It reduces the risk of overfitting, as it is more stable due to built-in regularization techniques such as bootstrapping and bagging, making it less susceptible to parameter selection. (3) While some machine learning algorithms (such as decision trees) are effective in understanding feature importance, they typically do not capture complex interactions between features as well as random forests do (31, 32). Moreover, the random forest algorithm strikes a good balance between model complexity and performance. Given these advantages, it has been widely applied in fields such as medicine, management, and public health (33, 34). Recent studies have also used this model to analyze issues related to the utilization of public health services among China’s migrant population (35). Therefore, building on similar studies, this research also uses the random forest model to measure the importance of various factors influencing health education for China’s migrant population.

Random forest algorithm consists of multiple decision trees, with the help of bagging algorithm to generate multiple training sets through random sampling of the sample. Decision trees serve as the base classifiers for each training set, with the final prediction determined by majority voting across trees. This approach is suitable for classification, regression, and prediction tasks. Importance analysis, on the other hand, takes the best variable selected in the decision tree as the classification node, so as to rank the importance of variables (36). The specific steps are as follows: (1) For each decision tree, the corresponding out of bag data (OOB) are selected to calculate the out of bag data error, which is recorded as err OOB1; (2) Randomly add noise interference to the feature X of all the samples of OOB (which can randomly change the value of the samples at the feature X), and then calculate the out-of-bag data error again, which is recorded as err OOB2; (3) Assuming that there are N trees in the forest, the importance of the feature X can be calculated by the following formula.


$$OOB\_\mathrm{store}=\frac{\overset{N}{\underset{i=1}{\sum }}\left(\mathrm{erroob}2i-\mathrm{erroob}1i\right)}{N}$$

This value can indicate feature importance because if the out-of-bag data accuracy decreases substantially (i.e., err OOB2 rises) after the addition of random noise, it indicates that this feature has a significant impact on the prediction results of the sample, which in turn indicates that the importance level is relatively high.

#### Binary logistic regression model

In this study, health education is a binary variable. Therefore, a binary logit model is used to analyze the impact of other factors on health education. The basic model is as follows:


$$\mathrm{logit}\frac{P_{i}}{1-p_{i}}=\beta_{0}+\beta_{1}X_{i}+\varepsilon_{i}$$

In which, $P_{i}$ denotes the probability that the mobile population will receive health education, $\beta_{0}$ is the intercept term, $X_{i}$ denotes other variables that will have an impact on health education, $\beta_{1}$ denotes the coefficient of influence of the relevant independent variable on health education, $\varepsilon_{i}$ is the error term.

#### Analysis of the concentration index

In research related to public health and health, methods for measuring inequality typically include the Concentration Index (CI), Gini coefficient, Oaxaca-Blinder decomposition, Lorenz curve, and range method, among others (37, 38). The Concentration Index, compared to other methods, has the following advantages (39–41): (1) The Concentration Index quantifies the tendency of health service utilization or health inequality, while the Gini coefficient only reflects the overall level of disparity. (2) The Concentration Index directly establishes a relationship between the relevant dependent variable (such as health education) and socioeconomic status, revealing structural inequalities, whereas the range method focuses solely on the distribution of the dependent variable itself. (3) The Concentration Index allows for decomposition techniques (such as Wagstaff decomposition) to attribute inequality to specific influencing factors. (4) Results obtained using the Concentration Index are not affected by the absolute level of the research indicators (e.g., through standardized Concentration Index), allowing for comparisons across populations, time periods, or countries. Currently, the Concentration Index is widely used not only to measure income-related inequality in the health sector but also to assess the equity of public health service utilization, medical service utilization, and health among China’s migrant population (42, 43). Based on the above analysis, this study also employs the Concentration Index to analyze the equity of health education for China’s migrant population and measure the contribution of related influencing factors to the equity of health education. Its calculation formula is as follows:


$$CI=2cov\left(y_{i},R_{i}\right)/\mu$$

In which $y_{i}$ represents the outcome variable of public health service utilization, μ denotes the average level of the variable in the population, and $R_{i}$ denotes the fractional rank of the sample *i* in the income distribution. The value of CI ranges from (−1,1), CI > 0 indicates that there is pro-rich inequality in the outcome variable and CI < 0 indicates that there is pro-poor inequality in the outcome variable. The larger the absolute value of CI, the more sensitive the distribution of the outcome variable is to the level of income, indicating a greater degree of inequality.

In this study, the concentration index decomposition method proposed by Wagstaff (44) was used to decompose the factors that may affect the equity of public health service utilization. By examining each factor’s contribution to inequality after decomposition, the main sources of inequity was identified, which helps target specific factors for control or elimination.

The decomposition formula is as follows:


$$C=\sum_{j}\left(\beta_{j}{\bar{X}}_{j}/\mu \right)C_{j}+GC_{\varepsilon }/\mu$$

C is the unstandardized concentration index, $\beta_{j},{\bar{X}}_{j},C_{j}$ are the regression coefficient (replaced by the marginal effect), mean and concentration index of the influence factor *j*, respectively, $\beta_{j}{\bar{X}}_{j}/\mu$ denotes the magnitude of the contribution of the influence factor j to the inequality in health, $GC_{\varepsilon }$ is the concentration index of the residual term, and μ is the mean value of the outcome of the utilization of the health services (i.e., the dependent variable).

### Statistical methods

**Figure 2:**
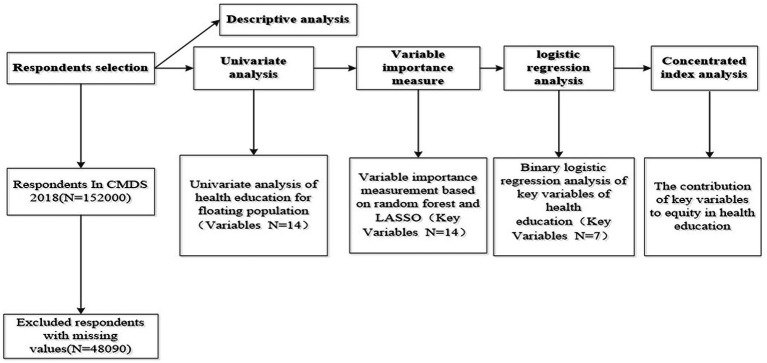
The flow chart of the study.

Data were organized and analyzed using Stata22.0 and RStudio software. Count data were expressed as the number of cases and percentage (%), and *χ*^2^ test was used for one-way analysis. Random forest model analysis was performed using RStudio software, and variables with statistically significant differences in the unifactorial analysis were included in the random forest model to derive and rank the variable importance scores. LASSO analysis was used for variable selection, and the screened variables were used for multifactorial analysis using binary logistic regression analysis. Finally, the decomposition of concentration index was used to identify the contribution of important variables to equity in health education. The flow chart is shown in Figure 2.

## Results

### Basic characteristics of the respondents

Table 2 shows the descriptive statistics of the main variables. Among 103,910 respondents, 86.08% report being healthy and 11.63% report having a basic health status. Regarding health education, the percentage of mobile population receiving health education is higher at 81.89%. In terms of health records, only 32.42% of the mobile population establish health records locally, and 67.58% still do not have health records. For family doctor services, only 15.09% sign up with local family doctors, while the percentage of those who do not sign up is 84.91%. Educational attainment among the mobile population is generally low, with only 19.91% holding university degrees or above, and 80.09% having high school education or below. Regarding household registration, 67.60% are registered as rural residents and 32.40% as urban residents. In terms of mobility, the proportion of inter-provincial mobility is the highest, followed by intra-provincial inter-city and intra-city inter-county mobility. In terms of income, the majority of the mobile population have below-medium income levels (62.85%). Regarding health insurance, 69.86% are covered by health insurance in their place of domicile, while 30.14% are covered by health insurance in their place of inflow.

**Table 2:** Distribution of respondents’ characteristics.

Variable		Quantity	Proportions (%)
Health status	Unhealthy	2,384	2.29
	Basic health	12,080	11.63
	Health	89,446	86.08
Health education	No	18,821	18.11
	Yes	85,089	81.89
Availability of health records	No	70,226	67.58
	Yes	33,684	32.42
Whether the family doctor signed up	No	88,235	84.91
	Yes	15,675	15.09
Genders	Female	50,899	48.98
	Male	53,011	51.02
Age	15–30	29,641	28.53
	31–45	48,358	46.54
	46–60	21,396	20.59
	≥61	4,515	4.35
Educational attainment	Illiterate	2,542	2.45
	Elementary school	14,179	13.65
	Middle school	43,614	41.97
	High school	22,890	22.03
	University and above	20,685	19.91
Marital status	Not in marriage	16,618	15.99
	In marriage	87,292	84.01
Working conditions	No occupation	17,723	17.06
	Employed	86,187	82.94
Household registration	Rural	70,248	67.60
	Urban	33,662	32.40
Range of mobility	Cross-county within city	18,533	17.84
	Cross-city within province	35,347	34.02
	Cross-province	50,030	48.15
Reasons for mobility	Family	14,985	14.41
	Work	87,036	83.76
	Other	1,889	1.82
Household income ranking	Lowest (<20%)	22,866	22.02
	Lower (20–39%)	21,207	20.41
	Medium (40–59%)	21,216	20.42
	Higher (60–79%)	20,038	19.28
	Highest (≥80%)	18,563	17.89
Insurance location	Place of domicile	72,588	69.86
	Place of inflow	31,322	30.14
Whether one is sick or not	No	91,544	88.10
	Yes	12,366	11.98

### Univariate analysis of health education for the mobile population

The results of the univariate analysis with whether the mobile population receives health education as the dependent variable are shown in Table 3. Statistically significant differences in health education are observed across various demographic characteristics including genders, ages, education levels, marriages, jobs, household registration status, mobility ranges, reasons for mobility, incomes, places of insurance coverage, illnesses, availability of health records, whether the family doctor signed up and health levels.

**Table 3:** Differences in health education for mobile populations across baseline characteristics.

Variable		Health education				*χ^2^*	*p*
		No		Yes			
		*n*	%	*n*	%		
Genders	Female	9,001	47.82	41,989	49.24	12.365	<0.001
	Male	9,820	52.18	43,191	50.76		
Age	15–30	4,561	24.23	25,080	29.48	747.923	<0.001
	31–45	8,172	43.42	40,186	47.23		
	46–60	4,869	25.87	16,257	19.42		
	≥61	1,219	6.48	3,296	3.87		
Educational attainment	Illiterate	757	4.02	1785	2.10	1.1e+03	<0.001
	Elementary school	3,528	18.75	10,651	12.52		
	Middle school	8,288	44.04	35,326	41.52		
	High school	3,343	17.76	19,547	22.97		
	University and above	2,905	15.43	17,780	20.90		
Marital status	Not in marriage	2,671	14.19	13,947	16.39	55.496	<0.001
	In marriage	16,150	85.81	71,142	83.61		
Working conditions	No occupation	3,956	21.02	13,767	16.18	255.154	<0.001
	Employed	14,865	78.98	71,322	83.82		
Household registration	Rural	13,491	71.68	56,757	66.70	174.348	<0.001
	Urban	5,330	28.32	28,332	33.30		
Range of mobility	Cross-county within city	2,823	15.00	15,710	18.46	671.508	<0.001
	Cross-city within province	5,329	28.31	30,018	35.28		
	Cross-province	10,669	56.69	39,361	46.26		
Reasons for mobility	Family	3,021	16.05	11,964	14.06	62.338	<0.001
	Work	15,405	81.85	71,631	84.18		
	Other	395	2.10	1,494	1.76		
Household income ranking	Lowest (<20%)	4,942	26.26	17,944	21.09	290.686	<0.001
	Lower (20–39%)	3,878	20.60	17,329	20.37		
	Medium (40–59%)	3,721	19.77	17,495	20.56		
	Higher (60–79%)	3,140	16.68	16,898	19.86		
	Highest (≥80%)	3,140	16.68	15,423	18.13		
Insurance location	Place of domicile	14,412	76.57	58,176	68.37	492.533	<0.001
	Place of inflow	4,409	23.43	26,913	31.63		
Whether one is sick or not health level	No	15,841	84.17	75,703	88.97	399.049	<0.001
	Yes	2,980	15.83	9,386	11.03		
Whether the family doctor signed up	No	18,164	96.51	70,071	82.35	2.4e+03	<0.001
	Yes	657	3.49	15,018	17.65		
Availability of health records	No	16,428	87.57	53,744	63.16	4.2e+03	0.000
	Yes	2,339	12.43	31,345	36.84		
Health level	Unhealthy	746	3.96	1,638	1.93	622.733	<0.001
	Basically healthy	2,880	15.30	9,200	10.81		
	Healthy	15,195	80.73	74,251	87.36		

### Importance measurement of factors influencing health education for mobile populations

#### Random forest data selection

This study randomly divided the samples into a training set (80%) and a validation set (20%). In the model training, we employed a Random Forest classifier for the classification task. To optimize model performance, we utilized Randomized Search for hyperparameter tuning. Randomized Search CV performs 5-fold cross-validation by randomly selecting parameter combinations multiple times, and the parameter set that yields the highest accuracy score is selected as the final result. The range of parameter values and their definitions are shown in Table 4.

**Table 4:** Parameter ranges and descriptions for the random forest model.

Parameters	Definition	Range of values	Optimal value
n_estimators(ntree)	Specifies the number of decision trees to be built in the random forest	1–500	187
max_depth	Specifies the maximum depth of each decision tree to control tree growth	1–20	17
min_samples_split(mtry)	Specifies the minimum number of samples needed to split an internal node, which influences the model complexity	2–20	18
min_samples_leaf	The minimum number of samples required to be at a leaf node, controlling the minimum size of leaf nodes	1–10	8

**Figure 3:**
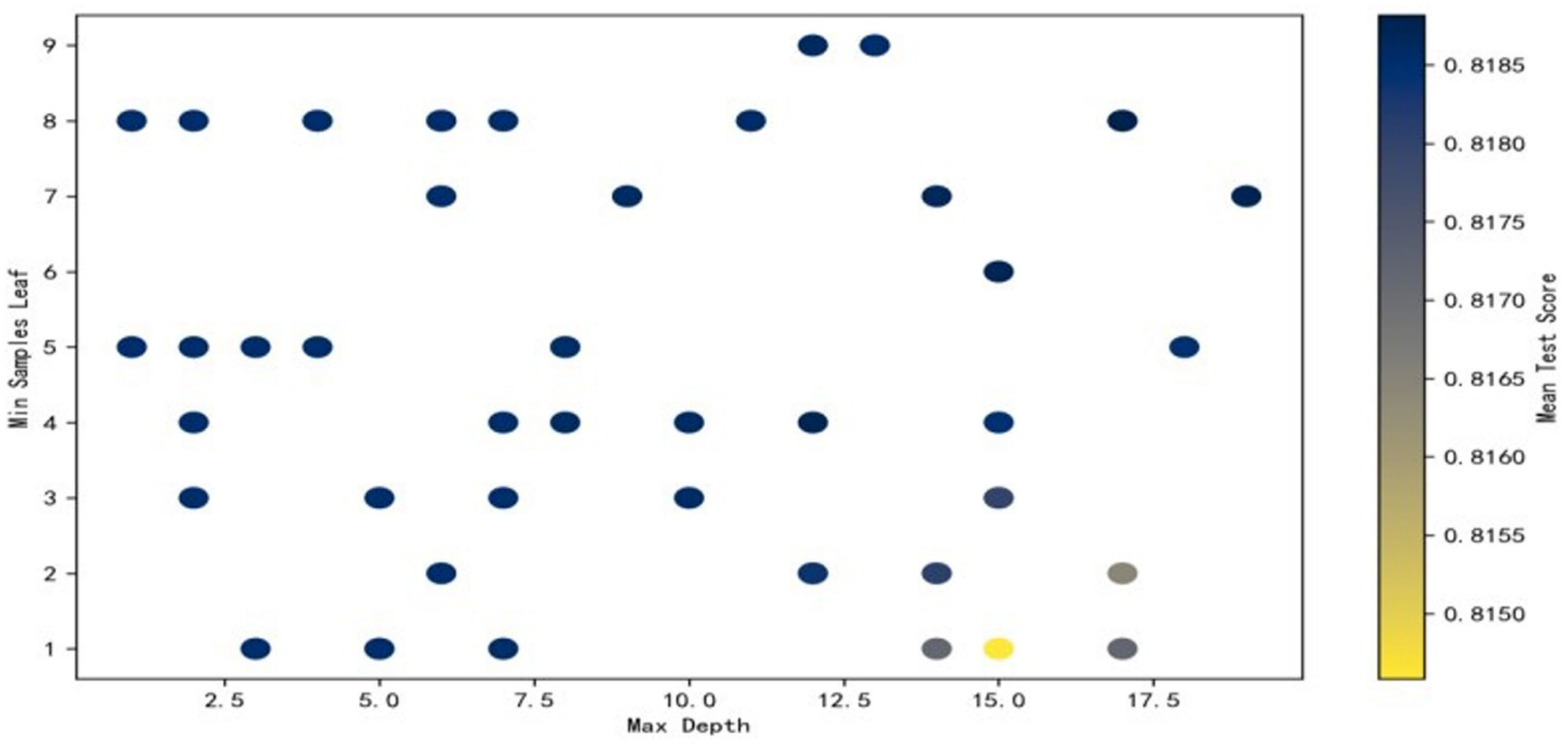
Randomized search process figure.

The process of Randomized Search is as follows: Due to the large number of parameters, we present the search process with the maximum depth on the x-axis and the minimum samples per leaf on the y-axis. The color of the scatter points represents the accuracy, with darker colors indicating higher accuracy. It can be observed that the point with a maximum depth of 18 and a minimum samples per leaf of 7 achieves the highest accuracy. This combination of parameters represents the optimal hyperparameters we were seeking (see Figure 3).

#### Ranking of importance of influencing factors

**Figure 4:**
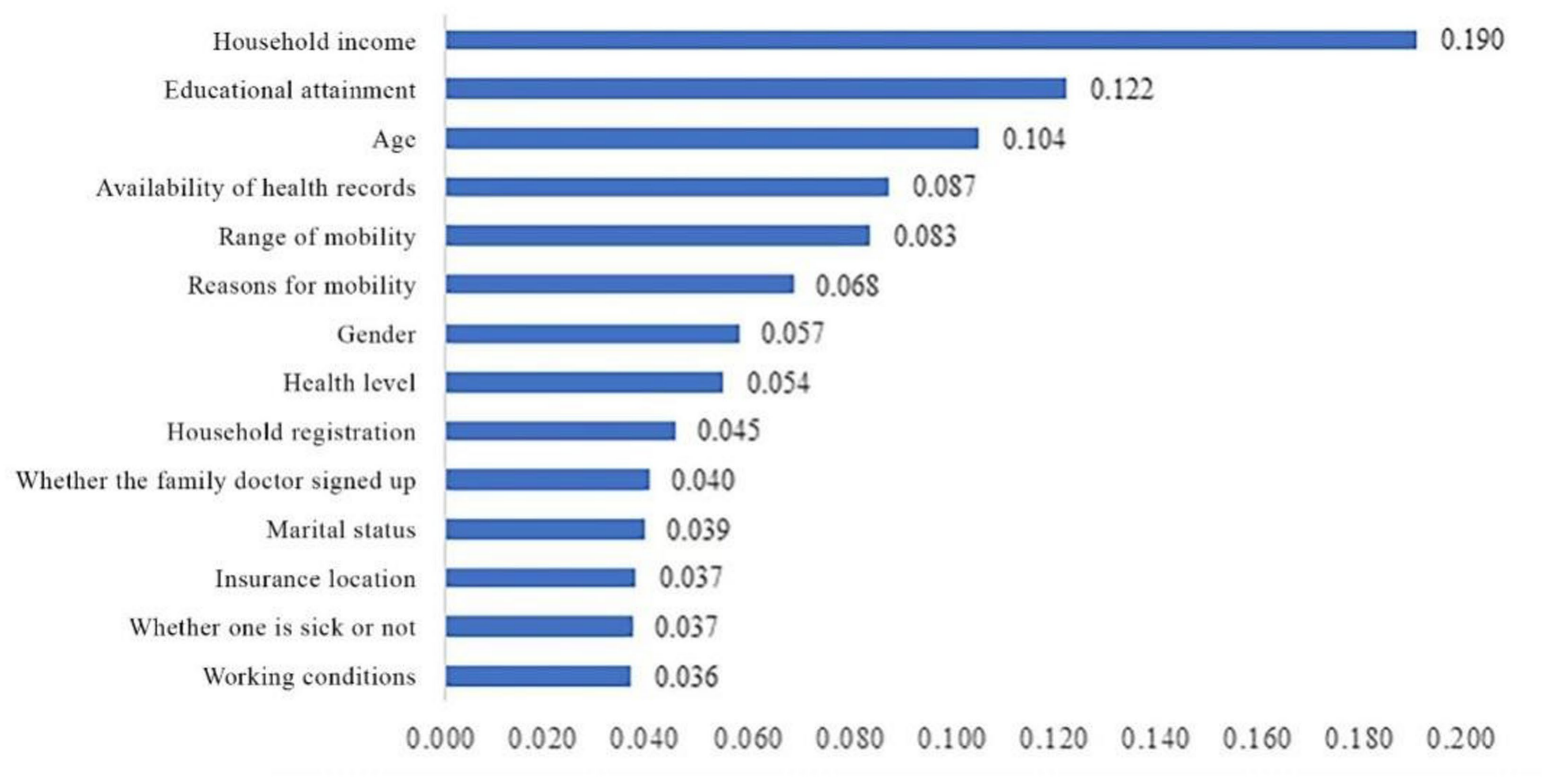
Ranking of importance eigenvalues of factors influencing health education for the mobile population.

Using health education as the dependent variable, variables with statistically significant differences in the one-way analysis were included in the random forest model. Using the Random Forest program package in RStudio to output the results, %Inc. MSE is the mean decrease in accuracy, and the larger the %Inc. MSE, the higher the importance of the variable in the influencing factors (45). Figure 4 shows the ranking of the importance of the factors influencing health education among the mobile population. In descending order of importance, these factors are: income, education, age, health record, range of mobility, reason for mobility, gender, health level, household registration status, contracted by the family doctor, marriage, place of insurance participation, illness, and work.

#### Characteristic variable screening

**Figure 5:**
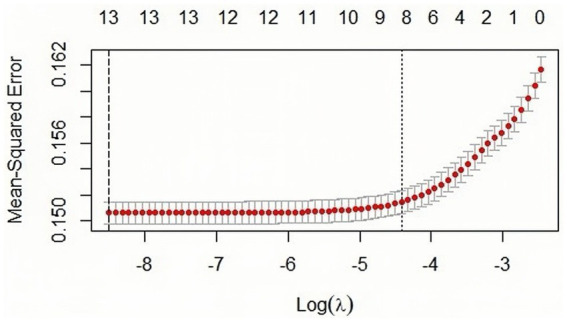
Characteristic variable screening based on LASSO analysis. The figure is based on the minimum criterion to filter the number of feature variables by plotting dashed lines at lambda.min (left dashed line) and lambda.lse (right dashed line), respectively.

**Figure 6:**
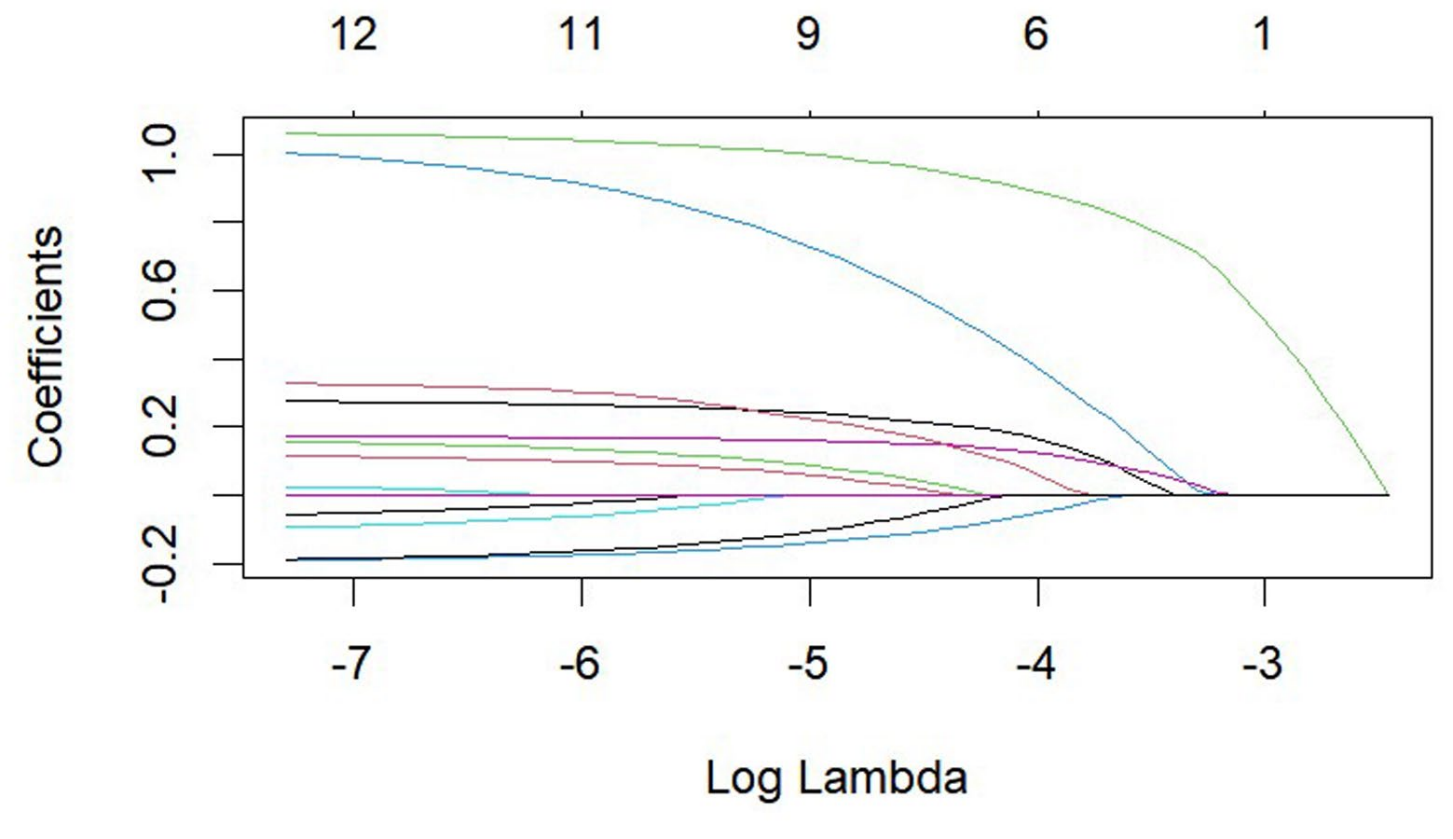
A total of 14 variables were included in this study and the figure shows 14 lines of different shades of color. Each curve represents the trajectory of the coefficient of each independent variable, with the vertical coordinate being the value of the coefficient and the lower horizontal coordinate being log(λ).

LASSO regression method achieves the feature selection and dimensionality reduction by setting unimportant feature coefficients to zero through L1 regularization. This method can screen the variables in order to avoid the function of covariance and overfitting. Compared with the traditional stepwise regression, LASSO can process all independent variables simultaneously, enhancing model stability. In this study, due to the inclusion of numerous independent variables which are prone to multicollinearity problems (46, 47), the LASSO regression model is therefore used for variable selection, and the important variables are further included in the logistic regression model for analysis. The LASSO regression method in this study is implemented by using glmnet package in R, with 100 iterations and the optimal *λ* value determined through 10-fold cross-validation. By calculation, the λ corresponding to the smallest mean square error is 0.000677 (see Figure 5). Figure 6 shows the LASSO variable screening diagram, based on the results of this diagram, seven variables were selected, namely income, education, age, health record, mobility range, reason for mobility and gender.

### Regression analysis of factors affecting health education of mobile population

A binary logistic regression analysis was conducted using health education as the dependent variable and the top 7 variables in terms of importance screened by the random forest model as the independent variables. The results are shown in Table 5. The findings indicate that higher income levels are associated with increased access to health education compared to the lowest income group. As age increases, the propensity of mobile population to access health education decreases. In terms of educational level, an increase in educational level promotes the mobile population’s access to health education compared to those who did not receive any health education. The mobile population with health records is more likely to receive health education compared to those who do not have health records (OR = 1.413, *p* < 0.001). Compared with intra-city cross-county mobility, intra-provincial cross-city and inter-provincial mobility was more likely to receive health education, indicating that the propensity of the mobile population to obtain health education increases as the scope of mobility expands. Compared with those who moved for family reasons, those who moved for other reasons (e.g., study, cross-country retirement, marriage, etc.) were more likely to receive health education (OR = 1.327, *p* < 0.001). The male mobile population was more likely to receive health education compared to the female mobile population (OR = 1.087, *p* < 0.001).

**Table 5:** Binary logistic regression analysis of influencing the acceptance of health education by the migrant population.

Variables		*β*	*p*	OR	95% CI
Household income ranking	Lowest (<20%) (reference group)				
	Lower (20–39%)	0.140	<0.001	1.150	1.096–1.208
	Medium (40–59%)	0.151	<0.001	1.163	1.107–1.221
	Higher (60–79%)	0.229	<0.001	1.257	1.192–1.324
	Highest (≥80%)	0.088	0.002	1.092	1.034–1.153
Age	15–30 (reference group)				
	31–45	−0.038	0.069	0.962	0.923–1.003
	46–60	−0.280	<0.001	0.756	0.719–0.794
	61-	−0.557	<0.001	0.573	0.529–0.629
Educational attainment	Illiterate (reference group)				
	Elementary school	0.143	0.004	1.155	1.048–1.272
	Middle school	0.369	<0.001	1.447	1.317–1.588
	High school	0.629	<0.001	1.876	1.699–2.072
	University and above	0.628	<0.001	1.873	1.690–2.076
Availability of health records	NO (reference group)				
	Yes	1.413	<0.001	4.108	3.924–4.301
Range of mobility	Cross-county within city (reference group)				
	Cross-city within province	0.428	<0.001	1.535	1.466–1.606
	Cross-province	0.432	<0.001	1.541	1.486–1.597
Reasons for mobility	Family (reference group)				
	Work	0.051	0.403	1.052	0.934–1.184
	Other	0.283	<0.001	1.327	1.185–1.485
Genders	Female (reference group)				
	Male	0.083	<0.001	1.087	1.052–1.123

### The influence of key factors on the equity of health education for mobile populations

#### Equity analysis of health education

**Figure 7:**
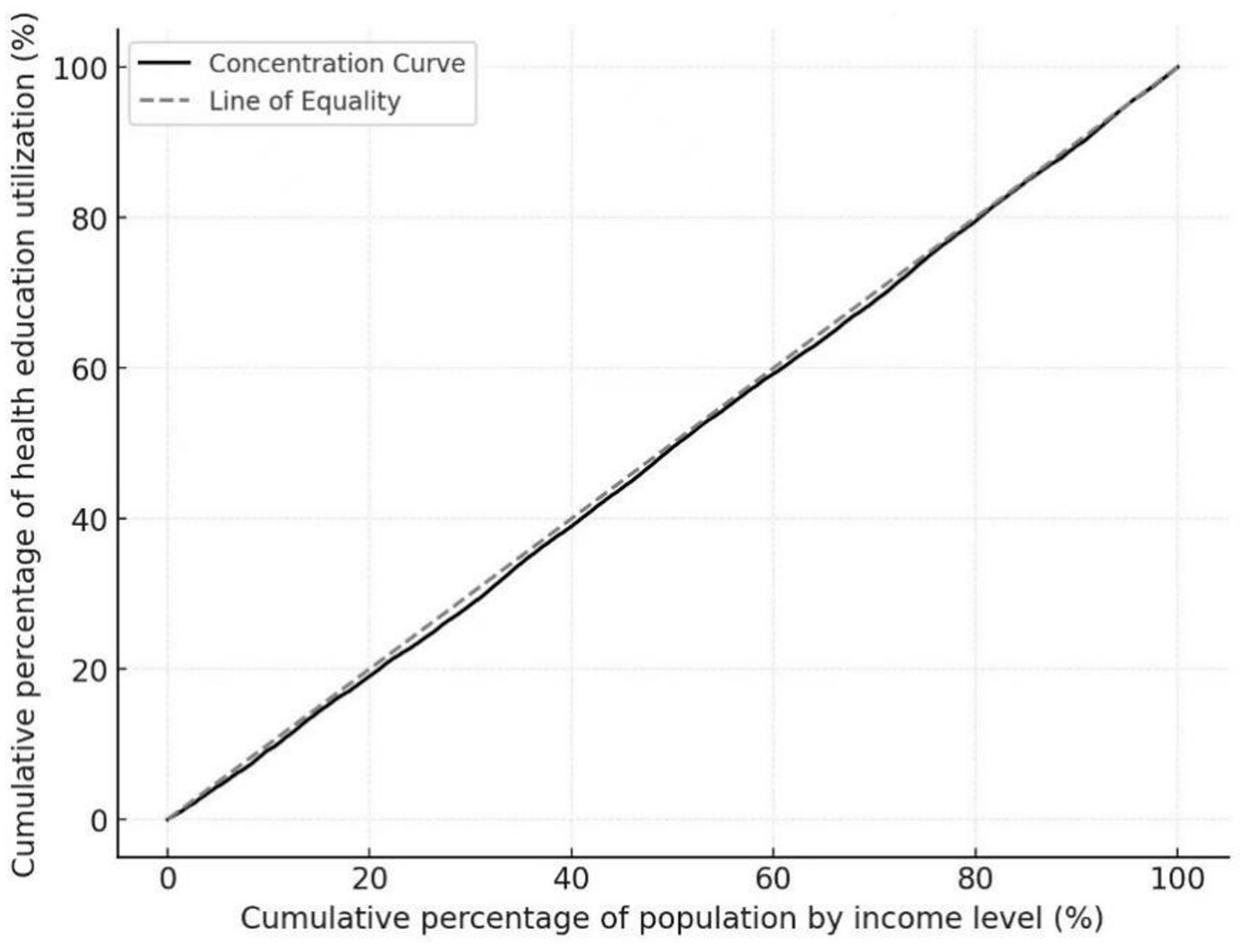
Concentration curve of health education service utilization.

The concentration index of health education for the mobile population is CI = 0.0121. The concentration index is greater than 0, and the concentration curve is below the absolute fairness line, which indicates that the distribution of health education service utilization is inclined to the population with higher income level, (see Figure 7).

#### Analysis of the degree of contribution of key factors to health education

The important variables of the seven health education influencing factors selected by LASSO were used as independent variables, and the health education was used as the dependent variable. The contribution of key factors to health education for the migrant population is further explored. The results are shown in Table 6. From the results of the decomposition of the concentration index, the effects of income, age, education level of junior high school and below, health records, range of mobility, reason for mobility and gender on the health education are all positive, indicating that the above factors increase the inequality of health education among the mobile population. In contrast, education levels of high school and above show negative concentration index for health education, suggesting that higher education levels help reduce health education inequality.

**Table 6:** Concentration index decomposition of the equity in health education service utilization among the mobile population.

Variable		Coefficient of elasticity	Concentration index	Contribution margin	Contribution rate
Household income ranking	Lowest (<20%) (reference group)				
	Lower (20–39%)	0.514	0.205	0.105	8.684
	Medium (40–59%)	0.554	0.199	0.111	9.156
	Higher (60–79%)	0.841	0.177	0.149	12.302
	Highest (≥80%)	0.323	0.192	0.062	5.136
Age	15–30 (reference group)				
	31–45	−0.096	0.004	−0.001	−0.033
	46–60	−0.708	0.016	−0.011	−0.926
	61-	−1.410	0.019	−0.028	−2.318
Educational attainment	Illiterate (reference group)				
	Elementary school	0.622	0.012	0.008	0.617
	Middle school	1.604	0.005	0.008	0.663
	High school	2.735	<0.001	−0.002	−0.023
	University and above	2.730	−0.012	−0.032	−2.663
Availability of health records	NO (reference group)				
	Yes	0.546	0.004	0.002	0.180
Range of mobility	Cross-county within city (reference group)				
	Cross-city within province	1.239	0.015	0.019	1.557
	Cross-province	1.251	0.011	0.013	1.075
Reasons for mobility	Family (reference group)				
	Work	0.119	0.012	0.001	0.122
	Other	0.665	0.029	0.019	1.599
Genders	Female (reference group)				
	Male	0.053	0.013	0.001	0.056

## Discussion

The purpose of this study was to analyze the key influencing factors of health education for mobile populations and their contribution to the equity of health education. This evidence from China not only enriches the theoretical understanding of the utilization of public health services among the floating population, but also provides practical support for improving health education policies for this group. Ultimately, it contributes to the dual objectives of achieving equality in the utilization of public health services and improving health outcomes for the floating population. The findings reveal that the proportion of mobile populations with a healthy status was 97.71%, indicating high overall health levels. However, since health forms the foundation for better utilization of human capital advantages (48), continued improvement in mobile populations health remains necessary. Health education is an important means of maintaining and improving health (49), and the results of existing studies also indicate that health education in the form of personal behavior change, policy and regulations, economic support, and organizational action can promote individuals’ acquisition of health knowledge and skills, which in turn can lead to changes in health behaviors. The results of existing studies have also shown that health education in the form of personal behavior change, policies and regulations, economic support, and organizational action can promote individuals’ acquisition of health knowledge and skills, which in turn can change health behaviors. While this study found that 81.89% of the mobile population received health education, 18.81% still lacked access. This falls significantly short of China’s target of 95% health education coverage for mobile populations by 2020, suggesting generally low health education coverage and levels among mobile population. These findings indicate a need for strengthened health education.

Regarding the influencing factors of health education for the mobile population, the results of this study show that health education is influenced by a variety of factors, which is similar to the results of previous studies (12, 50). However, this study advances the understanding by employing a random forest model to quantify the importance of various influencing factors. The analysis reveals varying degrees of importance among different characteristic factors affecting health education in mobile populations. The main factors that have significant influence on health education are income, age, education level, health record, scope of mobility, reason for mobility and gender. Certainly, the identification of these key factors is based on the evaluation of the 2018 CMDS cross-sectional survey data. In the future, with the availability of long-term longitudinal data, further analysis could be conducted to explore the trends of various influencing factors on health education for the floating population over time, thereby effectively identifying the most critical factors influencing health education within a long-term timeframe.

The study reveals that higher income levels facilitate better access to health education among mobile populations. This correlation may be attributed to higher-income mobile populations having both greater financial capacity for disease prevention and treatment, and enhanced health awareness. These groups can acquire more health information through health education, thereby maintaining or improving their health status (51). However, the analysis shows that most of the mobile population’s income is below the middle level. Therefore, health education initiatives should prioritize low-income mobile populations, such as further expanding the coverage of medical insurance for low-income people to reduce the cost burden of health education for such people (52), establishing personalized health education files for low-income people, and continuing to follow up on the changes in their health.

The impact of age on health education shows that acceptance rates decline as age increases. This trend may be attributed to older adult individuals having limited access to health education channels, while younger and middle-aged populations can access health education through new media platforms such as the internet and mobile phones (53, 54). Therefore, health education programs for mobile populations should develop age-specific content that addresses the unique health needs of older adult individuals. Examples include increasing awareness of prevention and treatment strategies for common chronic diseases among the older adult, which can enhance their interest in understanding and acquiring health-related knowledge. Furthermore, government agencies and community organizations can offer free health knowledge lectures and educational campaigns, and recruit healthcare professionals as volunteers to provide face-to-face health services to the older adult mobile populations.

Education level also has a significant effect on the acquisition of health education, which is also consistent with the results of existing studies (14). Education level objectively reflects the ability of individuals to obtain health information, learn health knowledge and master health skills (55). Higher education levels not only increase mobile populations’ health awareness but also provide access to more diverse channels for receiving health education. However, this study indicates that the education level of the mobile population is generally low, with the proportion of mobile people with high school or less education about four times higher than the proportion with university and above. The low level of education may lead to the inability of the mobile population to accurately and objectively evaluate their own health status, which may cause them to neglect the importance of health education. Therefore, future health education content should be simple, easily understandable, and appealing to target groups. Additionally, health education promotion methods should be optimized by leveraging the convenience and immediacy of mass media platforms, such as WeChat and websites, to stimulate mobile populations’ engagement in health education and enable faster, better access to health knowledge.

The establishment of health records also helps the mobile population to obtain health education. This may be because health records contain comprehensive health information including disease status and treatment history, making it easier for individuals to understand their health conditions and obtain individualized, targeted health education based on their needs. Moreover, mobile populations with established health records may demonstrate stronger health awareness and better compliance with basic public health service policies and organizational initiatives. However, this study found that only 32.42% of mobile populations have established health records, indicating insufficient utilization of basic public health services. Therefore, improving the health education of the mobile population, we should actively promote the establishment of health records, continuously improve the management of health records, strengthen the management and dynamic monitoring of the health data for the mobile population, and provide regular health services to them. This will maximize the role of health records in promoting health education.

In terms of the scope of mobility, this study indicates that as the scope of mobility expands, the mobile populations are more likely to have access to health education. This finding differs from those of Wang et al. on the utilization of public health services for access to the older adult mobile population. The results of this study indicated that older adult mobile populations moving within city limits had higher rates of health education acceptance and health record establishment compared to those moving across provinces (56). The reason for this disparity may be that the smaller the migration span, the stronger the stability, and the greater the reliance on medical service resources in the destination area. The differing results in this study may be related to the differences in the research subjects included.

Regarding reasons for mobility, populations moving for work, education, or retirement are more likely to receive health education compared to those moving for family reasons. This may be because populations migrating for reasons such as work, study, or relocating for older adult care are often in formal and structured environments, which provide more opportunities for health education. In contrast, those migrating for family reasons tend to focus more on family matters and may have less exposure to public health resources and educational activities. Furthermore, this research result also indicates that beyond individual social characteristics, factors related to mobility patterns also warrant attention. As China’s mobile population continues to grow in size and mobility frequency increases, the impact of mobility-related factors on health education will become increasingly significant. Therefore, strengthening health education for the mobile population should include increasing health knowledge training for family members, such as offering family health education courses for parents and caregivers in migrant families to help them promote the health of family members in the home environment. Additionally, the construction of a health information platform for the mobile population should be accelerated, and cross-regional resource and information sharing should be realized as soon as possible in order to improve the continuity of the utilization of public health services for the mobile population.

Gender is also a significant factor influencing health education. This is both similar to and somewhat different from the results of previous studies. For example, the study by Liping et al. concluded that women had better health education (13), while the results of Wang Xiaohui et al. showed that a higher percentage of men received health education compared to women (56). This study concluded that the reason for the difference in health education service utilization between genders is that the health status of women is generally higher than that of men, and health differences affect the health awareness and public health service utilization of mobile populations of different genders (57).

This study found that the seven key variables selected through LASSO contribute to increasing health education inequality among the mobile population, which is consistent with the findings of related studies (18). Further analysis of the contribution of important factors to the equity of health education reveals significant disparities in health education service utilization among mobile populations, with a bias toward high-income groups. This is also consistent with previous studies on the equity of public health service utilization among the mobile population in China. For example, relevant research shows that the concentration index for inpatient service utilization among China’s mobile population is 0.154, indicating a significant pro-rich inequity in the utilization of inpatient services (43). Furthermore, the study by Deng Yuhua et al. suggests that the utilization of family doctor contract services among China’s mobile population is more likely to benefit high-income groups (58). Additionally, the research by Zheng Cihui et al. reveals an inequity in the establishment of health records for China’s mobile population (59). Therefore, there is still a need to strengthen public health education for low-income mobile populations. Future efforts should focus not only on increasing overall health education acceptance rates among mobile populations, but also on addressing inequities in health education utilization to achieve the goal of equalizing public health service access. Certainly, this study’s assessment of the equity of health education service utilization among the mobile population is based on cross-sectional data, which allows for an analysis of the static situation of health education distribution at a specific point in time. However, with longitudinal data spanning a longer period, it would be possible to analyze the dynamic changes in this distribution over time, thereby providing a comprehensive understanding of the distribution changes in health education from both static and dynamic perspectives.

## Conclusion

Based on the 2018 China Migrants Dynamic Survey (CMDS), this study analyzed factors affecting health education and equity among mobile populations. The main conclusions are as follows. Firstly, the acceptance rate of health education for the mobile population still needs further improvement. Secondly, the health education of mobile population is affected by multiple factors with varying degrees of impact. Income, age, education level, health record establishment, mobility range, reasons for mobility, and gender were identified as the seven most significant influencing factors. All these factors have significant negative amplifying effects on health education of the mobile population. Thirdly, significant inequalities exist in health education utilization, showing a gap between current reality and the goal of equalized public health services.

Based on these findings, future efforts to strengthen health education for mobile populations should implement differentiated strategies according to the varying impacts and significance of different factors. Government and health departments should increase resource allocation for health education targeting mobile populations, with particular attention to vulnerable groups such as those with low income, older adult individuals, those with lower education levels, those without established health records, and those with wide mobility ranges. The ultimate goals are to enhance health literacy and improve the overall health conditions of mobile populations through continuously enriching health education content, innovating education methods, and expanding communication channels. However, when implementing health education interventions, it is essential to consider the specific characteristics of the mobile population and address potential ethical issues or other factors from four aspects: the implementing parties, the intervention process, the target audience, and the evaluation of intervention effects. For instance, the implementers of health interventions should possess relevant health-related professional knowledge; participants in the intervention should be informed of the objectives, methods, and potential benefits of the health education intervention; and post-intervention, it is crucial to focus on the follow-up and support of the intervention’s effects. These considerations will enhance the mobile population’s willingness to participate in health education interventions, promote the smooth implementation of intervention programs, and ensure that the interventions achieve the desired outcomes.

### Limitations

This study had several important limitations. First, the research relied on secondary data from a public dataset (with the latest version released in 2018), which presented certain time lag issues. As newer data become available, future research can analyze new characteristics of health education among mobile populations using more current information. In addition, the data used in this study are collected from a large-scale cross-sectional survey, which explored the relationship between health education and key factors among the mobile population in China. However, we were unable to draw causal inferences from the existing cross-sectional data. In the future, with the availability of more comprehensive data, more recent datasets (such as the Chinese General Social Survey or the China Labor-force Dynamic Survey) or longitudinal research data can be used to further explore the relationship between various influencing factors and health education for the mobile population. Additionally, it will be possible to examine the temporal changes in the key factors affecting health education and the dynamic changes in the equity of health education service utilization. Second, Although the China Migrants Dynamic Survey (CMDS) is highly representative, it also has certain limitations, such as respondents’ subjective biases when answering the questionnaire and uneven coverage of survey areas. Third, factors affecting health are complex. When selecting the influencing factors on the health of the mobile population, this study may overlook other potential factors (e.g., type of disease, severity of disease, health service utilization, etc.). Future research needs to consider incorporating additional potential influencing factors into the analysis. Fourth, although this study combined random forest models and logistic regression methods to analyze factors affecting health education among mobile populations, which helped overcome the limitations of using a single method, it did not utilize other machine learning techniques such as decision trees or XGBoost. In the future, these methods could be considered for in-depth research on issues such as health education for the mobile population, thereby providing stronger evidence to support improvements in mobile populations’ health status.

## Data Availability

The datasets presented in this study can be found in online repositories. The names of the repository/repositories and accession number(s) can be found below: data supporting the results of this study came from the National Mobile Population Dynamics Monitoring Survey (CMDS). The datasets generated and/or analyzed during the current study are available in the official website (https://www.ncmi.cn/project/project-showProjectList.html?type=data&id=d3).
